# Bovine mastitis and antimicrobial resistance in Pakistan’s dairy sector: current status and future prospects

**DOI:** 10.1007/s11259-025-10951-1

**Published:** 2025-11-12

**Authors:** Nauman Zaheer Ghumman, Mieghan Bruce, Amanda Duarte Barbosa, Muhammad Ijaz, Jiaxin Peng, Jully Gogoi-Tiwari

**Affiliations:** 1 School of Veterinary Medicine, College of Environmental and Life Sciences, Murdoch University, Perth, WA Australia; 2 Centre for Biosecurity and One Health, Harry Butler Institute, Murdoch University, Perth, WA Australia; 3 Department of Veterinary Medicine, University of Veterinary and Animal Sciences, Lahore, Pakistan

**Keywords:** Pakistan, Dairy industry, Milk safety, Antibiotic stewardship, Antibiotic misuse, Economic impact

## Abstract

Bovine mastitis remains one of the most widespread and economically significant diseases affecting the dairy sector in Pakistan. Despite being a leading global milk producer, Pakistan’s dairy industry faces persistent challenges in controlling mastitis, particularly among smallholder farmers with limited access to veterinary care and diagnostic tools. Antibiotics are frequently used to manage mastitis, often without veterinary oversight, contributing to the growing concern of antimicrobial resistance (AMR). The widespread presence of *Staphylococcus aureus*, a biofilm-forming pathogen, further complicates treatment and accelerates resistance development. Pakistan has initiated efforts to address AMR through national action plans. However, gaps remain in surveillance, responsible antibiotic use, and access to affordable alternatives. This review examines the current landscape of bovine mastitis in Pakistan, including its epidemiology, economic impact, therapeutic practices, and AMR trends. It also outlines practical, low-cost recommendations for improving mastitis management and reducing inappropriate use of antibiotics, particularly in rural settings. Addressing these interconnected challenges through locally adapted, sustainable approaches is essential for improving animal health, milk safety, and long-term productivity in Pakistan’s dairy sector, whilst addressing the global challenge of AMR.

## Introduction

Pakistan is an agriculture-based country where the livestock sector plays a vital economic role, contributing nearly 14.63% to the national GDP and supporting millions of rural livelihoods (GOP 2024). With an estimated 57.5 million cows and 46.3 million buffalo, Pakistan is the fourth-largest milk producer globally (GOP 2024; Sattar 2020). Despite this high output, the dairy sector faces major challenges, most notably bovine mastitis, a prevalent inflammatory disease of the mammary gland (Shafeeq et al. 2021; GOP 2024; Lakhani et al. 2023). Mastitis is recognised globally as a persistent and evolving problem, requiring novel control approaches (Benić et al. 2018), as it significantly reduces milk yield and quality, and compromises animal health (Sharif et al. 2009).

Mastitis represents a considerable economic constraint on the dairy sector in Pakistan, particularly among smallholder farms, where poor hygienic practices, limited access to veterinary services, and inadequate disease control measures contribute to its high prevalence (Ghafar et al. 2020). *Staphylococcus aureus* (*S. aureus*) is the predominant pathogen, accounting for over 50% of cases in indigenous cattle and buffalo populations (Ghumman et al. 2023; Ijaz et al. 2023; Javed et al. 2021, 2024). This aligns with findings from other regions where microbiological monitoring confirms *S. aureus* as a leading mastitis pathogen (Cvetnić et al. 2016, 2021). Additionally, other pathogens, including *Escherichia coli* (*E. coli*) (Ahmad et al. 2021), *Streptococcus spp.* (Ali et al. 2021), and *Klebsiella spp.* (Jamal et al. 2024) frequently cause mixed infections, exacerbating the challenges in managing bovine mastitis in Pakistan.

Mastitis treatment usually involves antibiotics, which are often used inappropriately and even without veterinary consultations (Mohyuddin et al. 2020). The widespread, often indiscriminate use of antibiotics has contributed to the emergence and spread of antimicrobial resistance (AMR) in Pakistan’s dairy sector (Bilal et al. 2021; Saleem et al. 2013). This challenge is not unique to Pakistan, as international studies have also established clear links between antimicrobial use and resistance in mastitis pathogens (Kovačević et al. 2022). The effects go beyond animal health because resistant bacteria and their genes can contaminate the food supply, especially through raw or poorly processed milk, creating risks for human health through animal-to-human transmission (Mohsin 2019). The situation is worsened by the reliance on antibiotics classified as critically important for human medicine, coupled with a lack of regulatory oversight in food-animal antibiotic use (Farhan et al. 2024).

The interplay between bovine mastitis and AMR presents a significant challenge in Pakistan’s dairy sector. However, there is a notable knowledge gap regarding how these two issues interact and influence each other. Understanding this relationship is essential for improving mastitis management, reducing antibiotic misuse and mitigating AMR risks in Pakistan’s dairy industry. The aim of this review is to investigate the interconnections between bovine mastitis and AMR in Pakistan’s dairy sector, with the following objectives to: (i) examine the epidemiology of bovine mastitis across different regions of Pakistan; (ii) discuss the economic impact of bovine mastitis on Pakistan’s dairy industry; (iii) review existing management strategies for bovine mastitis in Pakistan; (iv) assess antimicrobial usage practices in mastitis management and their potential role in the development of AMR; and (v) review emerging alternative therapies aimed at mitigating AMR and promoting sustainable dairy farming practices in Pakistan.

## Methodology

A thorough search was conducted using PubMed, Web of Science, and Google Scholar databases to access peer-reviewed articles related to bovine mastitis in Pakistan. Key search themes were: frequency of disease (bovine mastitis), antimicrobial resistance, alternative therapies and economic impact. The keywords used were combined using Boolean operators as follows: (“dairy cattle” OR “dairy buffalo”) AND (“bovine mastitis” OR “udder infection” OR “subclinical mastitis”) AND (“Pakistan” OR “Punjab” OR “Sindh” OR “Baluchistan” OR “KPK”). These were further combined with specific focus area terms using “AND” such as (“antimicrobial resistance,” OR “antibiotic usage,” OR “drug-resistant pathogens,”) OR (“alternative therapy” OR “herbal treatment” OR “phytotherapy” OR “probiotics”) OR (“Economic Impact” OR “Economic analysis” OR “Economic Loss” OR “Economic Burden” OR “Financial Impact” OR “Financial Loss”). Furthermore, along with the core focus areas, studies addressing broader contextual themes, including dairy industry structure, milk production systems, antibiotic usage, risk factors and farm management trends in Pakistan, were also searched. Literature published in English between 2010 and December 2024 was included. Studies not originating from Pakistan, unrelated to bovine mastitis, non-English publications, reviews, case reports, and articles outside the study scope were excluded. Articles only from Pakistan were selected specifically for the following sections: prevalence of bovine mastitis, resistant pathogens, alternative treatment options and economic impact. In addition to peer-reviewed literature, supplementary data were retrieved from authoritative national and international sources, including the Ministry of Finance, Pakistan (URL:https://finance.gov.pk/survey_2024.html), Ministry of National Health Services Regulations and Coordination (MNHSRC) (URL: https://www.nhsrc.gov.pk/), Ministry of National Food Security & Research, Pakistan (URL: https://www.mnfsr.gov.pk), Food and Agriculture Organization (URL: https://openknowledge.fao.org/), Trade and Development Authority of Pakistan (URL: https://tdap.gov.pk/) and relevant government reports such as the Economic Survey of Pakistan 2019 to 2024. The prevalence rates reported in this review were extracted directly from the included studies. The studies offered prevalence data depending on their research, population, and sampling methods. No new prevalence measurements were conducted; data were compiled and reported as presented in the original studies.

## Dairy industry in Pakistan

### a) Milk production and regional contributions

**Fig. 1:**
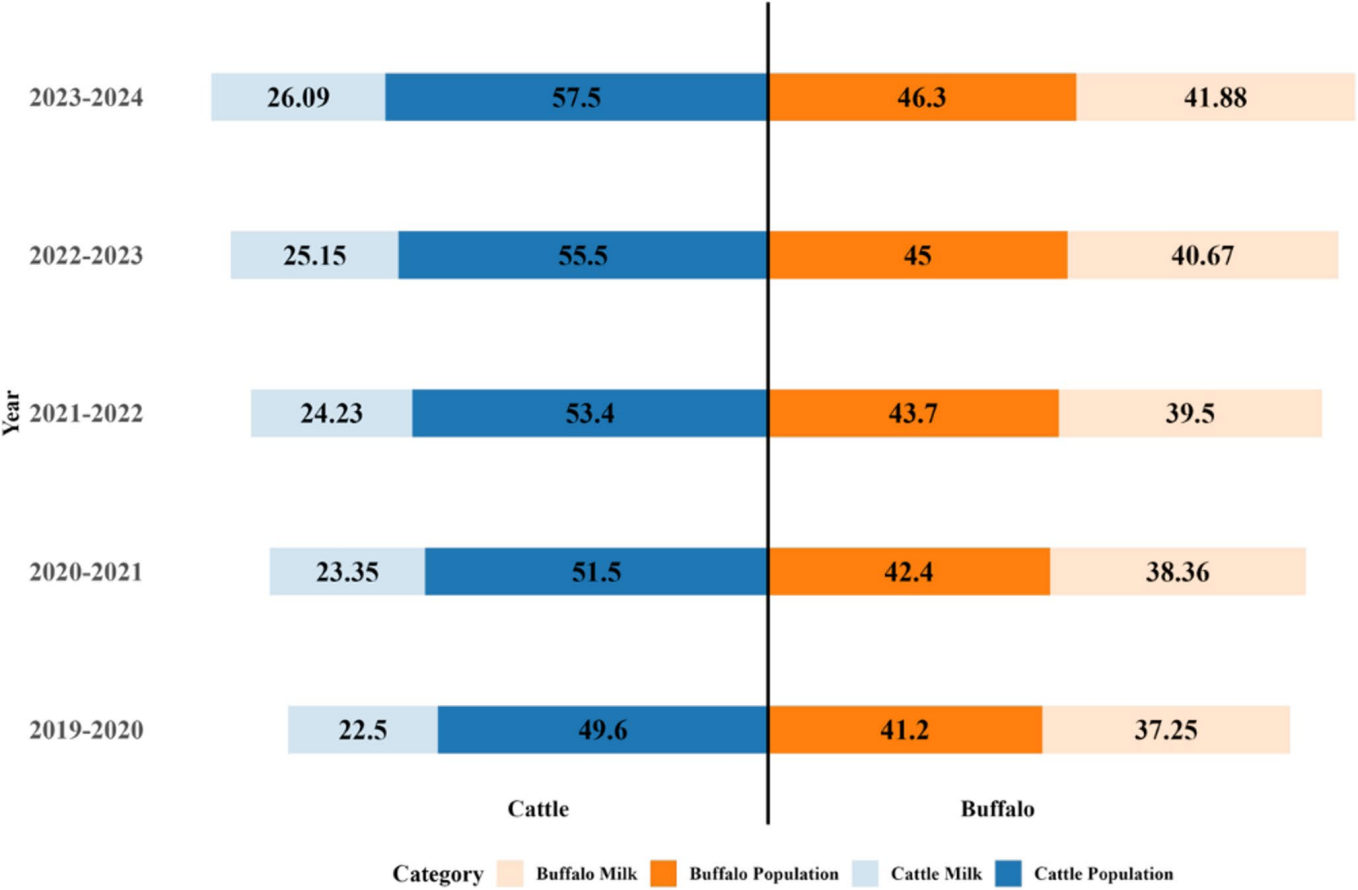
Bovine population (million animals) and milk production (million tons) in Pakistan: estimated trend over the last 5 years. (Source: “Economic Survey of Pakistan” 2023-2024 (GOP 2024). Note: blue indicates cattle data, and orange indicates buffalo data. Population is shown in millions (Darker blue and orange bars) and milk production in million tons (Light blue and orange bars). Data adapted from the “Economic Survey of Pakistan” 2023-2024 (GOP 2024)

Livestock plays a vital role in Pakistan’s economy, contributing 14.63% to the national GDP and 60.84% to the agriculture sector (GOP 2024). Within this sector, dairy farming forms a crucial component, supporting livelihoods and food security. The country possesses one of the largest dairy herds in the world, ranking ninth globally for its dairy bovine population (Bin Khalid and İsmet 2022). According to the Economic Survey of Pakistan (GOP 2024), the combined population of cattle and buffalo reached 103.8 million in 2023-24, showing consistent growth over the past five years (Fig. 1). This increase underscores the significance of herd expansion in meeting the rising demand for milk and related products.

**Fig. 2:**
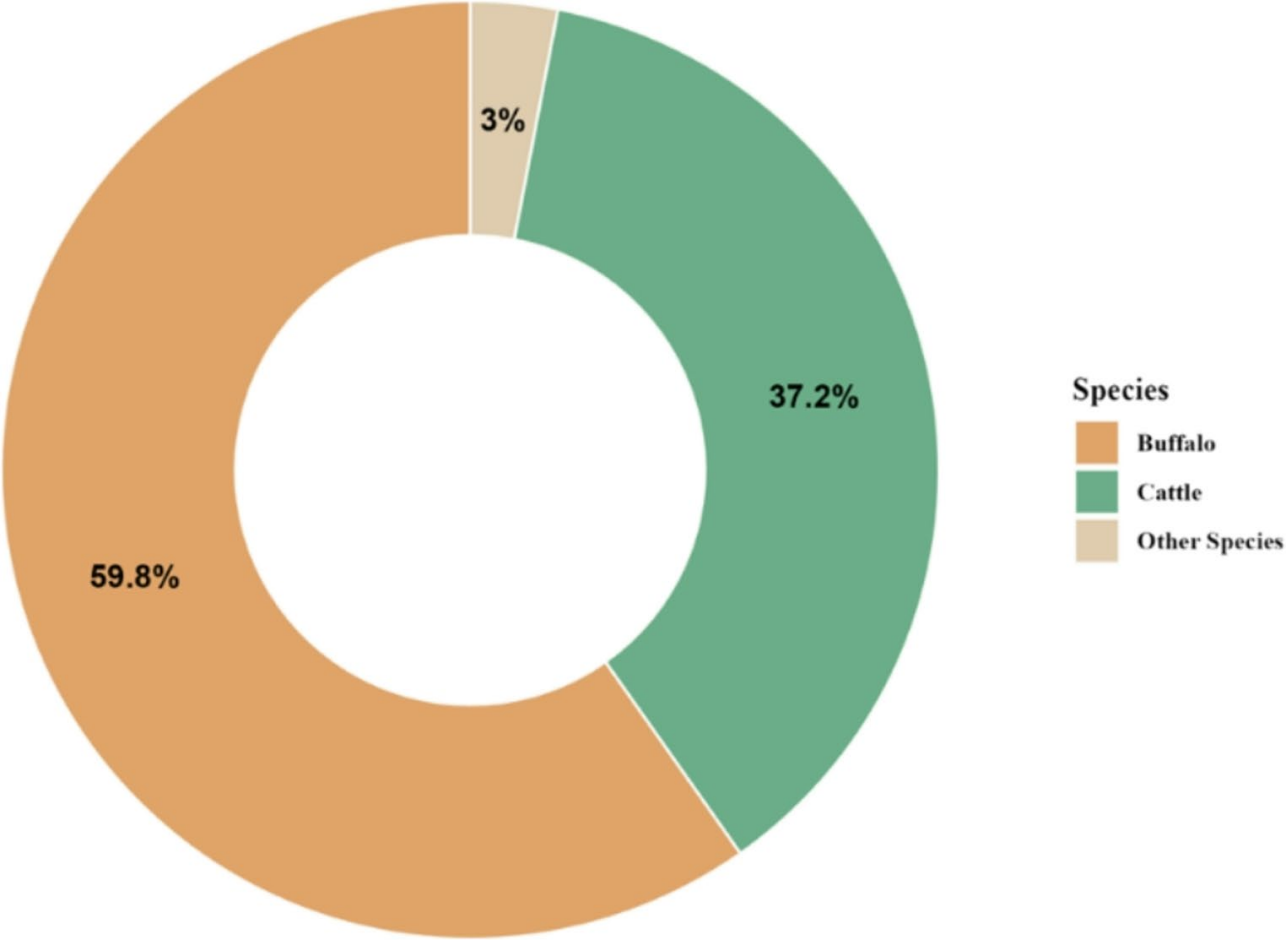
Milk production percentage distribution by species in Pakistan (2023-2024). Note: adapted from “Economic Survey of Pakistan” 2023-2024 (GOP 2024)

In line with herd growth, milk production in Pakistan has shown a steady upward trend, reaching an overall 70.07 million tons in 2023-24 (GOP 2024), making the country the fourth-largest milk producer globally (GOP 2024). Despite relatively lower productivity per animal (Tahir et al. 2019) the livestock sector recorded a 3.9% growth, the livestock sector recorded 3.9% growth, amounting to Pakistani rupees (PKR) 5,804 billion (GOP 2024). Species-wise, buffaloes contribute the largest share of milk production at 59.7% (41.8 million tons), followed by cattle at 37.2% (26.1 million tons), while the remaining 2.9% (2.1 million tons) comes from other dairy animals such as goats, sheep, and camels (Arshad et al. 2024; GOP 2024; Bin Khalid and İsmet 2022) as shown in Fig. 2.

**Fig. 3:**
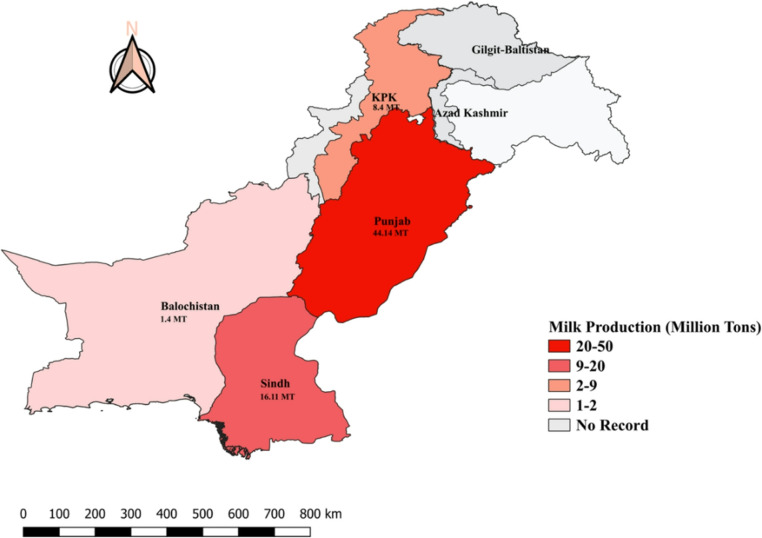
Major milk-producing regions (Provinces) in Pakistan. Note: Production (million tons) calculated from the total 70.07 Million Ton as per (GOP 2024)

Regionally, Punjab leads the country’s milk production, contributing 63% of the total output, followed by Sindh at 23%, Khyber Pakhtunkhwa at 12%, and Balochistan at 2% (TDAP 2023). Provincial contributions, highlighting Punjab’s dominant role in the national dairy economy, are illustrated in Fig. 3.

**Fig. 4:**
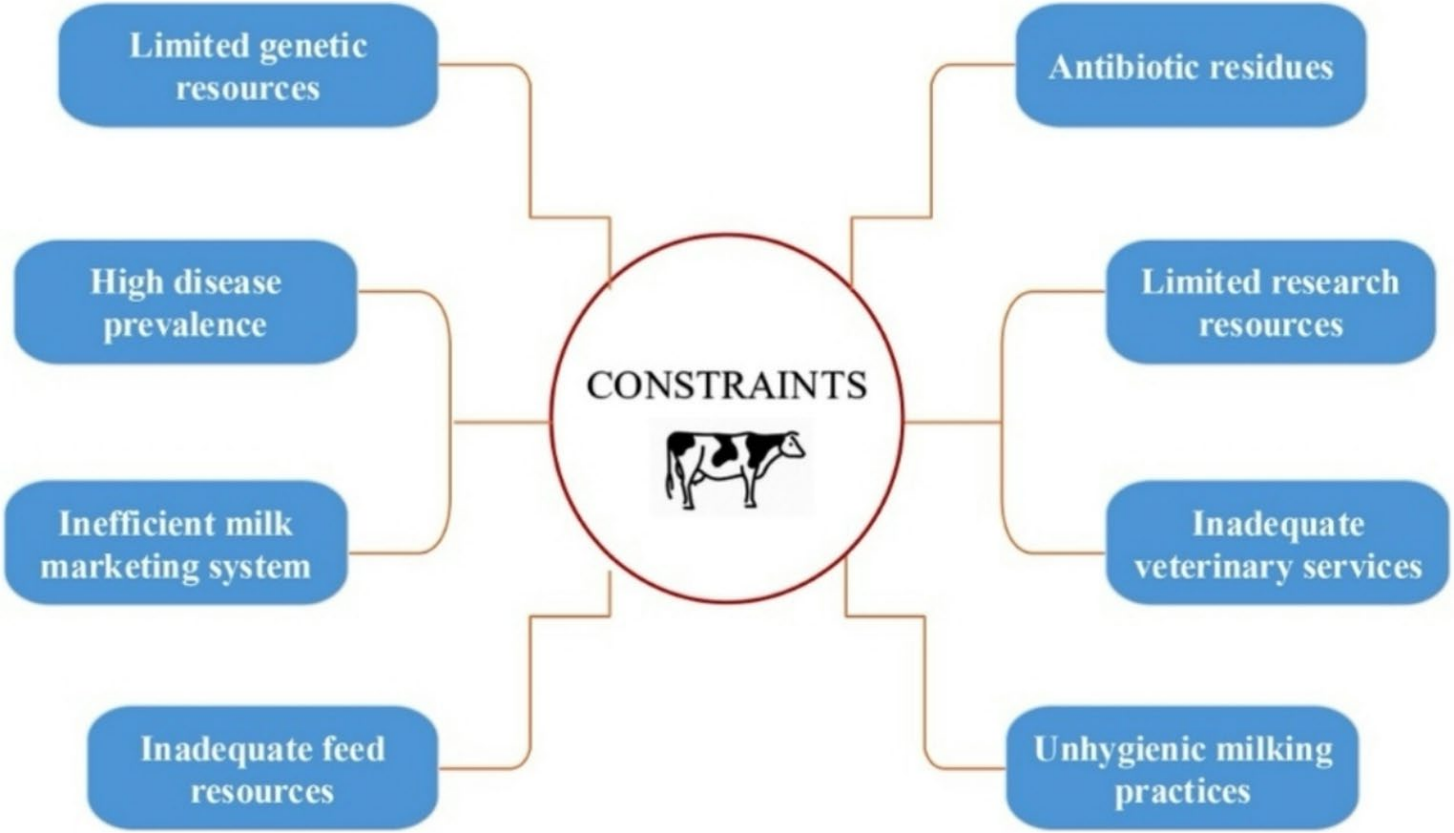
Key challenges and constraints in Pakistan’s dairy industry. Note: adapted from (Nadeem & Ahmad, 2024)

However, despite this vast scale of production, challenges such as bovine mastitis remain prevalent, among other factors, negatively impacting milk quality and economic returns across the sector (Fig. 4).

### b) Dairy farming systems and their contribution

Despite its significant economic role, dairy farming in Pakistan remains one of the least commercialised sectors (Bin Khalid and İsmet 2022). Pakistan has a diverse population of cattle breeds, including Sahiwal, Red Sindhi, Cholistani, Bhagnari, Dajal and Tharparkar, which are known for their adaptability to the local climate and resistance to diseases (Rehman et al. 2017; Dahlin et al. 1995). Additionally, exotic breeds such as Holstien-Friesian and Jersey are now being introduced more frequently due to their increased milk production (Khan 2022). Based on herd size, management practices, and milk production output, dairy farming is categorised into three systems: small farms, medium-sized farms, and corporate farms (Shahzad 2022).

#### Small farms

These farms account for approximately 70% of the dairy herds in Pakistan and hold less than 25 animals (Nadeem and Ahmad 2024; Saleem et al. 2013). Most farmers are landless and primarily keep dairy animals to meet household milk needs, producing on average 4–7 L of milk daily per animal. Surplus milk is either sold to local shops and milkmen at low rates or used for making traditional dairy products such as *desi ghee*,* cheese*, or *butter* (Bin Khalid and İsmet 2022; Shahzad 2022). However, challenges such as limited access to green fodder lead to the sale or slaughter of dry animals. Illiteracy and lack of training in modern farming practices further disadvantage these farmers, making them reliant on middlemen who control the milk marketing chain and offer low prices (Ziad et al. 2019). These factors also make small farms particularly vulnerable to diseases like mastitis, which may go misdiagnosed or inappropriately treated due to limited veterinary resources. This can lead to increased use of antibiotics, contributing to the growing issue of AMR (Ashfaq et al. 2015; Mohsin 2019).

#### Medium-sized farms

These farms typically have 25–200 dairy animals and are classified as rural commercial or peri-urban commercial farms (Farooq 2016). They often maintain a mix of local and imported high-yielding cattle breeds, each yielding up to 25 L per day at peak lactation (Shahzad 2022). Sometimes, they buy surplus cattle from corporate dairy farms or directly import high-producing exotic breeds. Milk is supplied to the market twice daily, either directly or through contracted retailers (Nadeem and Ahmad 2024). These farms use both concentrate and green fodder, but generally have limited awareness of proper feeding, farm management, disease control, and biosecurity. This limits production efficiency and results in inconsistent milk yields (Bin Khalid and İsmet 2022; Rehman et al. 2017). With limited access to modern veterinary care, medium-sized farms can face challenges with managing mastitis, which may be complicated by the overuse of antibiotics in treatment, thus contributing to AMR (Bin Khalid and İsmet 2022).

#### Corporate farms

Large-scale corporate farms house more than 200 up to thousands of dairy animals, primarily high-yielding exotic breeds. These farms are equipped with trained farm veterinarians and specialised staff. With advanced feeding strategies, including total mixed rations tailored to lactational stages, these farms produce 30–40 L of milk per cow per day during peak lactation. On average, these farms produce between 20,000 and 100,000 L of milk per day, depending on the herd size (Tahir et al. 2019). Unlike smaller operations, they maximise profits by processing, packaging and selling milk under their own brands (Shahzad 2022). Corporate farms are better equipped to manage animal health and disease. However, the intensive nature of their operations increases the risk of mastitis and antibiotic overuse, contributing to the growing issue of AMR (Hussain et al. 2018).

### c) Milk marketing and distribution systems in Pakistan

**Fig. 5:**
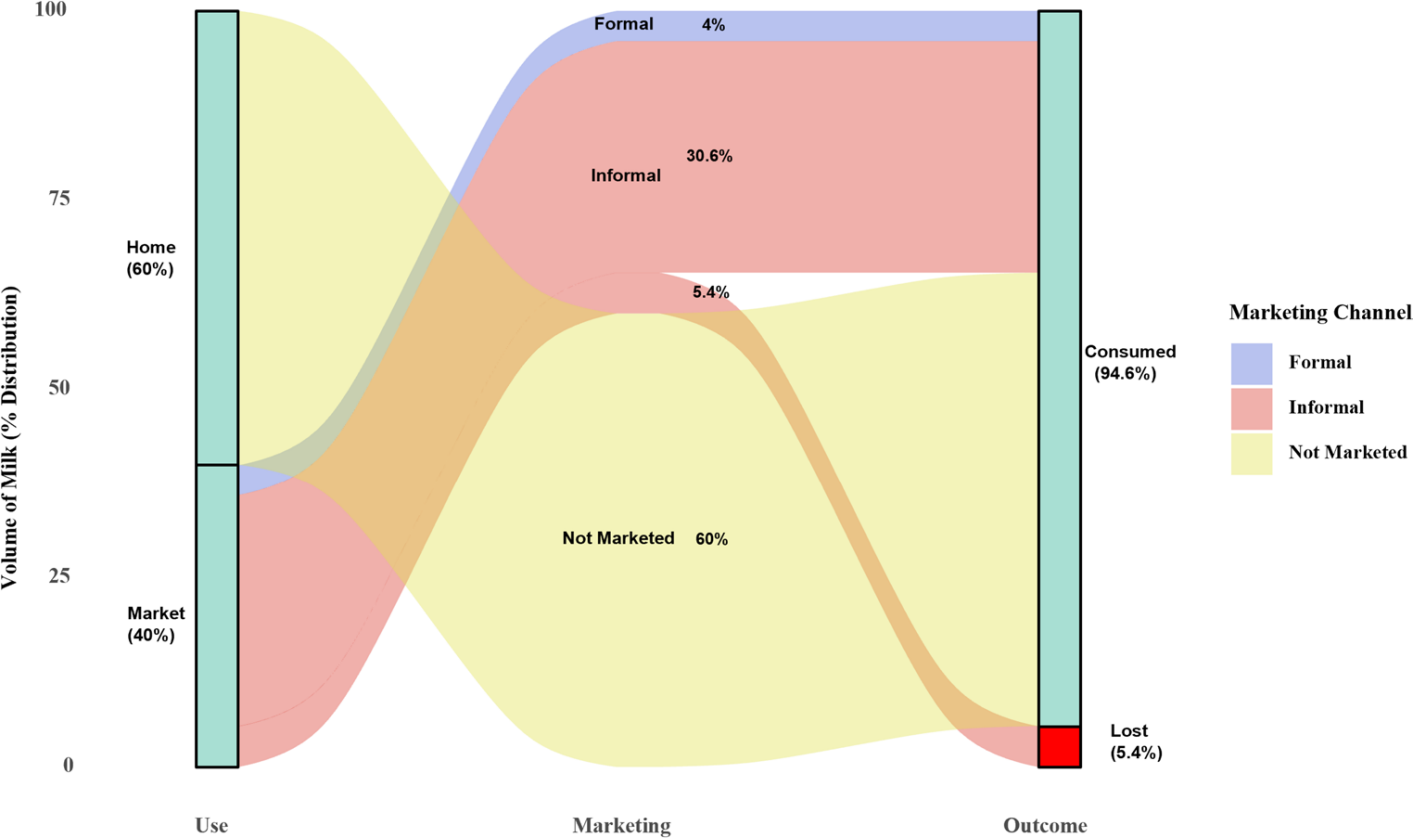
Milk marketing structure of small dairy farms in Pakistan

Pakistan’s milk marketing system is largely dominated by small-scale dairy farms. Around 60% of the milk produced is consumed at the household level and does not enter the commercial market (Fig. 5) (Bin Khalid and İsmet 2022). Of the remaining 40% that is marketed, approximately 30.6% of the total production is channelled through informal systems involving middlemen and local markets, such as milkmen (*dodhis*), traditional *halwais*, bakeries, milk shops, and tea stalls (Farooq 2016; Zia 2009). Only about 4% of the marketed milk reaches consumers through formal, regulated supply chains that include processing companies and commercial distributors (Bin Khalid and İsmet 2022). However, due to inadequate infrastructure for cooling, storage, and hygienic transport, the remaining 5.4% of milk is lost during transit before it reaches the end consumer (Fakhar et al. 2006) as shown in Fig. 5.

Recently, large commercial companies have entered the market and established milk collection centres and distribution junctions using insulated trucks and vans for better storage and distribution (Farooq 2016; Bin Khalid and İsmet 2022). Their milk collection centres are located at rural-urban junctions, and they purchase milk based on milk fat and total solids and have more efficient milk marketing and supply chains, including cooling tanks, chillers, and refrigerators, significantly improving milk quality, reducing post-harvest losses, and strengthening supply chains (Farooq 2016; Ziad et al. 2019). However, despite these advancements, the formal sector still struggles to compete with traditional informal networks, limiting its overall market penetration.

## Bovine mastitis overview

Bovine mastitis is a complex and economically important inflammatory condition of the mammary gland, commonly classified into clinical mastitis (CM) or subclinical mastitis (SCM) (Ashraf and Imran 2020; Cheng and Han 2020). Its severity depends on factors such as pathogen type, animal age, immune status, and stage of lactation (Kumar et al. 2020). CM is readily diagnosed by the swelling of the udder, redness, and abnormal milk consistency (Tiwari et al. 2013; Tomanić et al. 2023). In severe cases, cows may exhibit systemic symptoms, including fever, lethargy and anorexia (Kour et al. 2023). Severe CM is a leading cause of culling and on-farm mortality (Cheng and Han 2020; Haine et al. 2017), accounting for 26.6% of dairy cow deaths (Hagner et al. 2023). Cows with CM have a 40% higher culling risk, underscoring the disease’s economic and welfare implications (Haine et al. 2017). SCM is relatively difficult to diagnose because the infected cows seem normal with no apparent abnormalities of the udder (Cheng and Han 2020; Tomanić et al. 2023). Affected cows experience reduced milk production, high somatic cell counts (SCC) and altered milk composition (Kumar et al. 2020; Martins et al. 2020). Milk with SCC above 200,000 cells/mL is generally considered subclinically infected, and higher SCC is directly linked to lower milk yield and quality (Sharun et al. 2021). Studies also suggest that biochemical markers such as paraoxonase-1 activity and lipid profile may serve as potential diagnostic indicators for SCM (Kovačić et al. 2018; Nedić et al. 2019). Chronic SCM may decrease milk and total solids yields by approximately 24% and 22%, respectively (Martins et al. 2020). Feed additives have been explored as supportive strategies to mitigate SCM severity (Lamari et al. 2021).

Mastitis is a leading cause of economic losses in dairy herds worldwide (Kakooza et al. 2023). Each affected cow incurs an estimated annual loss of $147 USD due to reduced milk production and culling (Hogeveen et al. 2019). Over 70% of mastitis-related damage affects mammary tissue, significantly impairing milk production (Cheng and Han 2020; Liu et al. 2023). Globally, bovine mastitis-related losses are estimated at $22 billion USD, with $13 billion attributed to CM and $9 billion to SCM (Rasmussen et al. 2024). Asian countries bear the highest economic burden (Rasmussen et al. 2024). Comparative pharmacoeconomic analyses highlight how treatment choices, including antimicrobials and phytotherapy, impact overall disease costs (Kovačević et al. 2023). Additionally, studies indicate that mastitis prevalence and its effect on milk yield vary by breed and parity, underscoring its complex epidemiology (Gantner et al. 2024; Tomanić et al. 2024).

## Distribution and epidemiology of bovine mastitis in Pakistan

### a) Prevalence of bovine mastitis in Pakistan

Bovine mastitis is highly prevalent in Pakistan, with SCM occurring more frequently than CM (Table 1). SCM often goes undetected due to the absence of visible symptoms and thus remains in the herd for a prolonged period. In contrast, due to visible changes in the udder and milk, CM is more easily identified and treated, resulting in a lower prevalence (Javed et al. 2022). Here, prevalence refers to estimates from cross-sectional studies using SCM and CM diagnosis.

**Table 1:** Prevalence of subclinical and clinical mastitis in cattle and buffalo across different regions of Pakistan

Province	City/Region	Animal	Prevalence			Year	References
			CM (%)	SCM (%)	Total Samples		
Capital Territory	**Islamabad**	Cattle	-	54.0%	278	2024	(Shahzad et al. 2024)
		Buffalo	-	50.0%			
Punjab	**Rawalpindi**	Cattle	-	73.0%	278	2024	
		Buffalo	-	75.0%			
	**Faisalabad**	Buffalo	-	40.0%	249	2023	(Shahid et al. 2023)
	**Rawalpindi**	Cattle	1.8%	16.6%	432	2022	(Jalil et al. 2022)
	**Faisalabad**	Cattle	-	47.1%	385	2021	(Javed et al. 2021)
		Buffalo	-	44.7%			
	**Multan**	Cattle	-	38.0%	100	2021	(Ahmad et al. 2021)
	**Lahore**	Cattle	4.0%	56.0%	216	2021	(Imran et al. 2021)
		Buffalo	5.0%	55.0%			
	**Lahore**	Buffalo	-	55.5%	598	2020	(Hussain et al. 2020a)
	**Kasur**	Cattle	-	54.0%	90	2019	(Maalik et al. 2019)
		Buffalo	-	22.0%			
	**Pothohar**	Buffalo	-	67.3%	196	2019	(Khan et al. 2019a, b)
	**Bhimber**	Buffalo	8.0%	32.2%	1036	2018	(Hussain et al. 2018)
	**Cholistan**	Cattle	-	21.9%	1457	2016	(Qayyum et al. 2016)
	**Lahore**	Cattle	61.3%	30.6%	450	2012	(Mustafa et al. 2012)
		Buffalo	40.3%	59.6%			
	**Burewala**	Cattle	18.2%	33.6%	673	2012	(Hameed et al. 2012)
		Buffalo	24.6%	36.3%			
	**Muzaffargarh**	Cattle	-	36.0%	500	2011	(Bachaya et al. 2011)
	**Narowal**	Buffalo	-	48.0%	600	2011	(Ali et al. 2011)
	**Lahore**	Buffalo	-	40.6%	600	2011	
	**Okara**	Buffalo	-	42.0%	600	2011	
	**Sahiwal**	Buffalo	-	45.3%	600	2011	
	**Sahiwal**	Cattle	-	45.3%	500	2011	(Bachaya et al. 2011)
	**Muzaffargarh**	Buffalo	-	55.0%	300	2011	(Raza et al. 2011)
Khyber Pakhtunkhwa (KPK)	**Swat**	Cattle	-	31.0%	140	2023	(Khan et al. 2023)
		Buffalo	-	75.6%			
	**Hazara**	Buffalo	24.6%	75.3%	440	2022	(Javed et al. 2022)
	**Peshawar**	Cattle	21.2%	62.3%	1013	2022	(Ali et al. 2022b)
		Buffalo	17.8%	68.9%			
	**Province Wide**	Cattle	17.9%	69.0%	1166	2021	(Ali et al. 2022a)
		Buffalo	10.8%	79.4%			
	**Peshawar**	Cattle	81.0%	-	2791	2017	(Rafiullah et al. 2017)
		Buffalo	79.0%				
	**D.I. Khan**	Buffalo	13.6%	41.8%	-	2014	(Ali et al. 2014)
	**D.I Khan**	Buffalo	-	53.0%	300	2012	(Akhtar et al. 2012)
Sindh	**Khairpur**	Cattle	-	63.5%	400	2022	(Ruk et al. 2022)
	**Hyderabad**	Buffalo	-	54.2%	210	2016	(Baloch et al. 2016)
Balochistan	**Centre-West**	Buffalo	61.8%	38.1%	579	2022	(Saif et al. 2022)
	**North West**	Cattle	-	54.1%	144	2022	(Hameed et al. 2022)

Cattle generally exhibit a higher overall prevalence of mastitis than buffalo; however, regional variations exist. Across multiple studies in Pakistan, the average SCM prevalence is approximately 50.7% in cattle and 46.2% in buffaloes, while CM prevalence is 34.2% in cattle compared to 28.9% in buffaloes (Imran et al. 2021; Javed et al. 2021; Maalik et al. 2019; Shahzad et al. 2024). The higher prevalence in cattle is attributed to factors such as a soft udder sphincter (Imran et al. 2021) and the growing number of exotic and crossbred cattle (Khan 2022). Mastitis is more prevalent in these breeds than in the local breeds (Khan et al. 2015; Maalik et al. 2019) possibly due to their high milk yield potential and reduced adaptability to Pakistan’s climate (Khan et al. 2015).

Prevalence of mastitis across different regions in Pakistan is significantly different (Table 1), and is influenced by environmental factors, treatment regimes, management and pathogen diversity (Ali et al. 2011). Cities like Rawalpindi, Lahore, Faisalabad, Kasur and Muzaffargarh in Punjab report high prevalence of mastitis. In Lahore, SCM prevalence was 56% in cattle and 55% in buffaloes (Imran et al. 2021). In Rawalpindi, SCM prevalence was 73% in cattle and 75% in buffaloes (Shahzad et al. 2024). However, some studies report higher mastitis prevalence in buffaloes, including Faisalabad, with 40% (Shahid et al. 2023) and 44.7% (Javed et al. 2021) in 2023 and 2021, respectively. In Lahore (Mustafa et al. 2012), Burewala (Hameed et al. 2012), and Muzaffargarh (Raza et al. 2011), the prevalence of SCM was recorded as 59.6%, 36.3%, and 55%, respectively, in buffaloes. Similarly, studies from province KPK also show higher prevalence of mastitis in buffaloes as compared to cattle, where Swat (Khan et al. 2023), Hazara (Javed et al. 2022) and Peshawar (Ali et al. 2022b) were found to be the high prevalence regions. In contrast, the Southern and Central regions of Pakistan show relatively lower prevalence (Multan: 38% SCM in cattle; Cholistan: 21.9% SCM in cattle). Despite limited studies, available data from Sindh (Baloch et al. 2016; Ruk et al. 2022) and Baluchistan (Hameed et al. 2022; Saif et al. 2022) indicate SCM rates exceeding 50%, suggesting a substantial but under-recognised mastitis burden in these regions. Reported prevalence can be influenced by differences in study design, sample size, environmental conditions, farm management, milking hygiene, husbandry systems, and possible underreporting (Qayyum et al. 2016; Javed et al. 2022). These findings highlight the need for regional surveillance and standardised study designs to accurately assess the mastitis prevalence across Pakistan.

### b) Temporal dynamics of bovine mastitis prevalence

The prevalence of bovine mastitis in Pakistan has remained consistently high over the past decade, with significant regional and species variations. Here, prevalence refers to point-in-time estimates from cross-sectional studies that use SCM and CM diagnosis. While study methodologies vary, the data provide a broad picture of trends over time. The reported prevalence values are drawn from independent cross-sectional studies conducted on different dairy farms across various regions of Pakistan. Although these studies do not represent the same herds, they collectively illustrate temporal variations in the occurrence of SCM and CM in different regions of Pakistan. The estimated burden of SCM in buffaloes in Lahore increased gradually over time, rising to 59.6% in 2012 (Mustafa et al. 2012), compared with 40.6% in 2011 (Ali et al. 2011) and slightly decreased to 55.5% in 2020 (Hussain et al. 2020a) and 55% in 2021 (Imran et al. 2021). The prevalence of SCM in cattle has increased from 30.6% in 2012 (Mustafa et al. 2012) to 56% in 2021 (Imran et al. 2021), reflecting an increasing trend in cattle. Comparatively, CM in buffaloes reported 40.3% in 2012 (Mustafa et al. 2012) but decreased considerably to 5% in 2021 (Imran et al. 2021). CM in cows declined remarkably from 61.3% in 2012 (Mustafa et al. 2012) to 4% in 2021 (Imran et al. 2021). This indicates better CM management but persistent challenges with SCM.

In the Rawalpindi/Potohar region, the proportion of SCM in buffaloes rose to 75% in 2024 (Shahzad et al. 2024) compared to 67.3% in 2019 (Khan et al. 2019a, b). The cattle SCM increased dramatically in 2024, up to 73% (Shahzad et al. 2024), contrasting with its levels in 2022 (16.6%) (Jalil et al. 2022). However, the prevalence of CM in cattle was still low in 2022 (1.8%) (Jalil et al. 2022), which indicates an improvement in better detection and management of clinical cases.

In Faisalabad, SCM in buffaloes showed a slight decline from 44.7% in 2021 (Javed et al. 2021) to 40% in 2023 (Shahid et al. 2023), whereas cattle maintained a high prevalence at 47.1% in 2021 (Javed et al. 2021), pointing to ongoing subclinical infection concerns.

The KPK region exhibits fluctuating patterns. SCM in buffaloes declined from 53% in D.I. Khan (2012) (Akhtar et al. 2012) to 41.8% in 2014 (Ali et al. 2014), but subsequently increased to 79.4% in 2021 (province-wide) (Ali et al. 2022a, b) and 75.3% in Hazara (2022) (Javed et al. 2022). SCM in cattle remained high, with 69% in 2021 (Ali et al. 2022a, b) and 62.3% in 2022, reported in Peshawar (Ali et al. 2022a, b). CM in buffaloes ranged from 10.8% (2021, province-wide) (Ali et al. 2022a, b) to 24.6% in Hazara (2022) (Javed et al. 2022). Swat reported one of the highest SCM rates in buffaloes at 75.6% in 2023 (Khan et al. 2023), indicating persistent mastitis challenges in the province, despite some variations across regions.

In Sindh, SCM remains high, with 63.5% in cattle (Ruk et al. 2022) and 54.2% in buffaloes (Baloch et al. 2016), indicating persistent SCM over time. In Balochistan, SCM was 38.1% in buffaloes (Saif et al. 2022) and 54.1% in cattle (Hameed et al. 2022). CM in buffaloes was alarmingly high at 61.8% (Saif et al. 2022), highlighting a significant CM burden.

### c) Risk factors and pathogen diversity of bovine mastitis

**Fig. 6:**
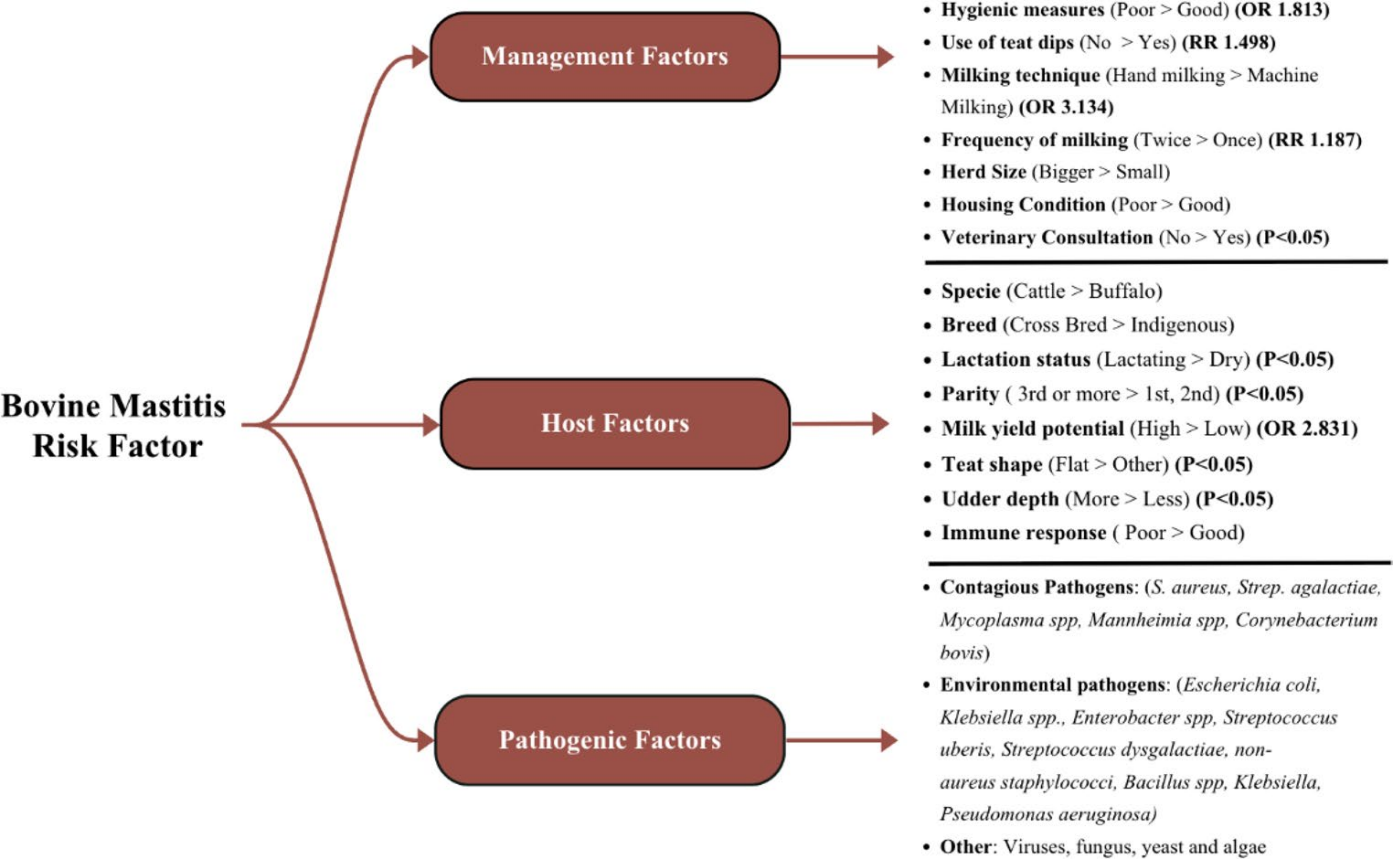
Risk factors contributing to bovine mastitis in Pakistan’s dairy industry. Note: OR (Odds ratios) and RR (Relative Risk) values indicate effect size; p-values are shown where effect sizes are unavailable. ">" indicates which group had a greater effect. Data source: management factors: (Javed et al. 2021), (Aqib et al. 2017), (Ghumman et al. 2023), (Hussain et al. 2018) host factors: (Shahzad et al. 2024), (Ghumman et al. 2023), (Javed et al.^2021^), (Javed et al. 2022), (Qayyum et al. 2016), (Khan et al. 2023). Pathogenic factors: (Shoaib et al. 2021)

Several intrinsic and extrinsic factors contribute to the susceptibility of dairy bovines to mastitis (Tezera and Aman Ali 2021). These include a combination of management, pathogen, and host-related factors that significantly influence the occurrence of bovine mastitis in Pakistan (Qayyum et al. 2016; Aqib et al. 2017; Javed et al. 2021; Malik and Verma 2017; Shoaib et al. 2021). The key host, environmental, and pathogen-related risk factors associated with bovine mastitis in the region are illustrated in Fig. 6.

#### Management-related risk factors

Poor milking hygiene and inadequate udder care during milking significantly increase mastitis risk, as dirty udders and legs serve as sources of infection (Javed et al. 2021). The absence of teat dipping facilitates bacterial spread between animals during milking (Aqib et al. 2017). Traditional hand milking methods are still widely used for Indigenous cattle and buffaloes and are associated with higher mastitis rates due to inconsistent pressure and increased contamination risks (Ghumman et al. 2023). Other contributing factors include increased milking frequency, large herd sizes, and indiscriminate use of antibiotics without a veterinary prescription. Such practices not only enhance pathogen exposure and increase disease transmission but also pose risks of AMR (Aqib et al. 2017; Ghumman et al. 2023; Hussain et al. 2018; Kleinlützum et al. 2013).

#### Host-related risk factors

Certain genetic and physiological traits predispose to mastitis. Crossbred and exotic dairy cows show higher incidence rates compared to local breeds, probably because of the insufficient adaptation to the climate conditions of the region and increased metabolic stress (Khan et al. 2015; Maalik et al. 2019; Shahzad et al. 2024). Lactating animals are at increased risk compared to dry animals, due to the higher stress of constant milk production and exposure to pathogens (Ghumman et al. 2023). Increased parity and milk yield also contribute to the susceptibility because repeated stress on the udder can interfere with udder immunity (Gantner et al. 2024; Hussain et al. 2013; Javed et al. 2021). Also, it depends on the udder and teat conformation. It has been found that animals that have flat or pointy teats are more susceptible to infection relative to cylindrical or round teats (Javed et al. 2022). The greater the depth of the udders, the higher the chances of contamination by contact with soiled bedding (Qayyum et al. 2016).

#### Pathogen diversity

In Pakistan, bacterial infections are the leading cause of bovine mastitis, with *S. aureus*,* Streptococcus agala*ctiae, and *E. coli* causing more than 75% of all mastitis cases (Shoaib et al. 2021). Several pathogens have been isolated from clinical and subclinical cases, including *Streptococcus spp.* (Ali et al. 2021; Hameed et al. 2008), E. *coli* (Ahmad et al. 2021; Ali et al. 2017; Aslam et al. 2021), *Mycoplasma spp.* (Ashraf et al. 2018; Saif et al. 2022), and, less commonly *Corynebacterium spp*., *Bacillus spp.*, and *Pseudomonas spp*. (Anjum et al. 2010). The most common is *S. aureus*, which is particularly virulent as it produces various virulence factors that enhance its pathogenicity and persistence in udder tissue (Vujinović et al. 2023). All these enable *S. aureus* to bind, colonise, and persist in mammary tissue, evading the host immune response (Shoaib et al. 2021). Its prevalence in Pakistan may also be governed by suboptimal milking practices, poor hygiene, limited awareness among farmers and the increasing antibiotic resistance (Ahmed et al. 2022; Ghumman et al. 2023; Jalil et al. 2022). Several studies have reported regional variations in *S. aureus*-associated mastitis prevalence. In the Potohar region, SCM cases in cattle and buffalo showed 23.5% and 18% *S. aureus*, respectively (Shahzad et al. 2024). Meanwhile, Ghumman et al. (2023) found in another research that *S. aureus* was prevalent in 28.7% of subclinical cases of dairy bovines in the Rawalpindi district. The *S. aureus* prevalence rate of 18.4% was observed in cases with SCM involving buffaloes in the Hazara district (Javed et al. 2022). In another study in Faisalabad, the prevalence in cattle and buffaloes was recorded at 37.1% (Javed et al. 2021). Moreover, a previous report from Kasur district reported *S. aureus* in 36.7% of the cattle and 25% in the buffalo cases of mastitis (Maalik et al. 2019).

### d) Economic impact of bovine mastitis in Pakistan

Mastitis is one of the most significant causes of financial losses in the dairy industry of Pakistan (Ashfaq et al. 2015). However, few studies have evaluated its full economic consequences. Most studies estimate only certain components of the total cost, lacking a comprehensive approach (Ashfaq et al. 2015; Fareed et al. 2017). A recent report by Ghafar et al. (2020) reveals that mastitis causes substantial economic losses to farmers, reducing milk production by 25%, and lowering animal value. Consistently, Thomas et al. (2002) reported an annual loss of approximately 200 million USD in Pakistan due to animal diseases. These losses are largely attributed to the high prevalence of mastitis, unhygienic milking practices and the widespread use of self-medication on small-scale dairy farms (Ghumman et al. 2023; Javed et al. 2021).

Farmers often rely on ethnoveterinary drugs because they perceive allopathic drugs as costly and time-consuming (Munibullah et al. 2024; Dilshad et al. 2010; Hussain et al. 2020b). Such unregulated or inappropriate treatment frequently results in chronic mastitis, further increasing economic losses (Ghafar et al. 2020). In another study in Karachi, Pakistan, the average economic loss per animal/day was estimated at 640 PKR (equivalent to USD 2.27) due to reduced milk production due to mastitis, which accounted for 17% of total economic losses in buffaloes (Fareed et al. 2017). In Faisalabad, Ashfaq et al. (2015) reported that the cost of mastitis was an average of PKR 2,183 (USD 7.76) per animal, with small farmers incurring PKR 3,925 (USD 13.95), medium farmers PKR 3,134 (USD 11.14), and large farmers PKR 1,817 (USD 6.46) for both cattle and buffalo mastitis. Milk loss was the largest cost component at PKR 1,890 (USD 6.72) per animal, compared to treatment costs of only PKR 293 (USD 1.04). These figures were derived from surveys conducted during the study period.

These findings highlight the disproportionate impact on small farmers, driven by poor farming practices, lack of mastitis management strategies, limited veterinary support in rural areas, and unregulated drug use (Hussain et al. 2012). In addition, Ashfaq et al. (2014) stated that even on large farms, the benefit-cost ratio (BCR) of mastitis control is less than 1, indicating that the costs of control often exceed the financial benefits. This indicates that mastitis represents a high-cost problem for farms of all sizes, and the issues are even more significant in small farms due to limited resources.

## Antibiotic use and antimicrobial resistance in Pakistan’s dairy sector

### a) Mastitis treatment and antimicrobial usage (AMU) in livestock production

In Pakistan, AMU in food-producing animals is high and is estimated to rise by 44% between 2020 and 2030 (Mulchandani et al. 2023). Significant knowledge and practice gaps exist regarding antibiotic usage, particularly for mastitis treatment among farmers (Farhan et al. 2024). This problem is further aggravated by veterinarians’ inadequate understanding of responsible antibiotic use principles (Saman et al. 2023). Several Knowledge, Attitude, and Practice (KAP) surveys have evaluated AMU behaviours of both veterinarians and farmers (Farhan et al. 2024; Saman et al. 2023). Farhan et al. (2024) reported that a substantial number of farmers do not use antibiotics appropriately to treat mastitis because 22% of them do not consult veterinarians, 39% do not complete a full course of antimicrobial therapy, 32.9% do not follow proper dosage recommendations, and 40% are not aware of the risk related to misusing the antibiotics. Alarmingly, 90% of farmers believe self-medication for mastitis is more economical, contributing to unregulated antibiotic use and increased AMR risk. A parallel survey of veterinarians revealed that 66% of veterinarians had never attended seminars on AMR, 71% lacked knowledge on detecting antibiotic residues, 60% prescribed antibiotics inappropriately for viral infections, and 60% admitted to administering double doses (Saman et al. 2023). These findings highlight an urgent need for improved veterinary education and stricter regulations to mitigate inappropriate antibiotic practices.

The livestock sector in Pakistan, particularly the dairy industry, is a major consumer of antibiotics (Mohsin and Umair 2020; Ullah 2023), with mastitis treatment contributing significantly to antimicrobial consumption (Lubna et al. 2023). Approximately 46% of Pakistan’s total veterinary antibiotic use (34% for treatment and 12% for feed) occurs in this sector, with annual veterinary antibiotic usage totalling 1,481.78 kg (Ullah 2023). On corporate dairy farms, Umair et al. (2020) documented the use of 138.34 kg of antibiotics in 2018, with aminoglycosides, penicillins, and tetracyclines being the most commonly used classes for mastitis treatment. Notably, 42.46% of all antimicrobials used were classified as “critically important” for human medicine by the World Health Organisation (WHO) (World Health Organization 2019), highlighting the potential risks for human health due to cross-species resistance. Another study analysed the import data from 2017 to 2019 and found that 805.39 tonnes of WHO-classified Critically Important Antimicrobials (CIA HtP) with the Highest Priority (CIA-HtP) were imported. The highest imported antimicrobials were tylosin (240.84 tonnes), enrofloxacin (235.14 tonnes), and colistin (219.73 tonnes) (Umair et al. 2022). This reflects a high level of antibiotic use, estimated at 10.05 mg/kg of animal biomass (Umair et al. 2022).

### b) Antimicrobial resistance

AMR poses a significant health challenge around the globe (Kovačević et al. 2022; Samreen et al. 2021), and low and middle-income countries (LMICs), such as Pakistan, bear a disproportionately high burden (Murray et al. 2022). AMR-related deaths remain high among human populations, and the rising AMU among livestock, especially in dairy farming, has become an emerging issue because of its role in spreading resistant pathogens (Mohsin and Umair 2020).

It is especially alarming in the dairy industry, where antibiotics are widely used both for treating mastitis and for preventing the disease (Lubna et al. 2023). Several studies have reported multidrug-resistant (MDR) pathogens associated with bovine mastitis in Pakistan, including *Escherichia coli* (Ullah et al. 2022), S. *aureus* (Haq et al. 2024; Khan et al. 2022; Lubna et al. 2023), *S. agalactiae* (Leghari et al. 2023), and *Klebsiella pneumoniae* (Jamal et al. 2024; Saddam et al. 2023). Mastitis-causing bacteria have also been reported to show resistance to critically important antimicrobials (CIAs) such as cephalosporins, fluoroquinolones, and aminoglycosides, and this raises significant concerns about treatment failures and the risk to public health (Haq et al. 2024; Lubna et al. 2023). Table 2 describes the most important MDR pathogens isolated in mastitis cases in Pakistan between 2019 and 2024.

**Table 2:** Prevalence of antibiotic-resistant pathogens isolated from bovines in Pakistan

Drug-resistant Pathogen	Specie	Method of Detection	Isolated	Prevalence	Sample size	Year	References
Multidrug resistant ***S. aureus*** (MDRSA)	Cattle	Phenotypic	SCM	50.0%	543	2024	(Haq et al. 2024)
	Cattle	Phenotypic	Raw milk	20.0%	150	2023	(Lubna et al. 2023)
	Buffalo	Phenotypic	CM	80.0%	1250	2022	(Khan et al. 2022)
Methicillin resistant ***S. aureus*** (MRSA)	Cattle and Buffalo	Genotypic (*mecA*)	SCM	21.0%	278	2024	(Shahzad et al. 2024)
	Cattle	Genotypic (*mecA*)	SCM	25.9%	518	2024	(Ijaz et al. 2024)
			CM	16. 4%	172		
	Cattle	Genotypic (*mecA*)	SCM	17.0%	543	2024	(Haq et al. 2024)
	Cattle and Buffalo	Genotypic (*mecA*)	SCM	30.0%	249	2023	(Shahid et al. 2023)
	Cattle	Genotypic (*mecA*)	SCM	45.1%	58	2023	(Khan et al. 2023)
	Buffalo	Genotypic (*mecA*)	SCM	50.0%	82		
	Cattle	Genotypic (*mecA*)	Raw Milk	81.8%	150	2023	(Lubna et al. 2023)
	Buffalo	Genotypic (*mecA*)	SCM	19.6%	440	2022	(Javed et al. 2022)
	Cattle	Genotypic *(mecA)*	SCM	52.1%	173	2022	(Ghumman et al. 2023)
	Buffalo	Genotypic *(mecA)*	SCM	42.1%	172		
	Cattle	Genotypic *(mecA)*	SCM	24.1%	384	2022	(Muzammil et al. 2023)
	Buffalo	Genotypic (*mecA*)	SCM	17.0%	384		
	Buffalo	Genotypic (*mecA*)	SCM	18.8%	516	2022	(Ijaz et al. 2023a)
	Buffalo	Genotypic (*mecA*)	SCM and CM	20.0%	22	2020	(Khan et al. 2020)
	Cattle	Genotypic (*mecA*)	SCM	54.0%	104	2019	(Khan et al. 2019a, b)
	Cattle and Buffalo	Genotypic (*mecA*)	SCM	74.3%	900	2018	(Aqib et al. 2018)
	Cattle and Buffalo	Genotypic (*mecA*)	SCM	34.0%	900	2017	(Aqib et al. 2017)
Vancomycin resistant ***S. aureus*** (VRSA)	Cattle and Buffalo	Genotypic (*vanB*)	SCM	10.9%	768	2022	(Muzammil et al. 2023)
	Cattle and Buffalo	Phenotypic	SCM	12.6%	385	2021	(Javed et al. 2021)
Tetracycline resistant ***S. aureus*** (TRSA)	Cattle	Genotypic *(tetK)*	SCM	46.8%	543	2024	(Haq et al. 2024)
	Cattle	Genotypic (*tetK*)	Raw Milk	63.6%	150	2023	(Lubna et al. 2023)
	Buffalo	Genotypic *(tetA)* *(tetB)* *(tetC)*	CM	*tetA (*52.5%) *tetB (*51.4%) *tetC* (46.4%)	1250	2022	(Khan et al. 2022)
Beta-lactam resistant ***S aureus*** (BRSA)	Cattle	Genotypic *(blaZ)*	SCM	55.3%	543	2024	(Haq et al. 2024)
	Buffalo	Genotypic *(blaTEM)* *(BlaSHV)* *(blaCMY-*2)	CM	*blaTEM* (63.9%) *BlaSHV* (42.1%) *blaCMY-*2 (50.7%)	1250	2022	(Khan et al. 2022)
	Buffalo	Genotypic *(blaZ)*	SCM/CM	40.0%	22	2020	(Khan et al. 2020)
Aminoglycoside resistant ***S. aureus*** (ARSA)	Cattle	Genotypic *(aacA-aphD)*	SCM	13.8%	543	2024	(Haq et al. 2024)
Erythromycin-Resistant ***S. aureus***	Buffalo	Genotypic (*ermA*,* ermB*,* ermC*)	SCM/CM	*ermA* (53.1%) *ermB* (21.4%) *ermC* (35.7%)	19	2022	(Rasool et al. 2022)
Sulphonamide resistant ***S. aureus***	Buffalo	Genotypic *(Sul1)* *(Sul2)* *(Sul3)*	CM	*Sul1* (49.6%) *Sul2* (42.1%) *Sul3* (48.9%)	1250	2021	(Khan et al. 2022)
Multi-drug resistant ***Klebsiella pneumonia***	Cattle	Phenotypic	CM	44. 4%	700	2023	(Saddam et al. 2023)
Multi-drug-resistant extended spectrum β-lactamase Producing-***Klebsiella pneumonia*** (MDR ESBL)	Cattle	Genotypic *(blaSHV)* *(blaTEM)* *(blaCTX-M)*	SCM	*blaSHV* (56.0%) *blaTEM* (36.0%) *blaCTX-M* (30.0%)	700	2024	(Jamal et al. 2024)
Extended Spectrum ***B-***Lactamase Shiga Toxin-Producing ***E.Coli*** (ESBL STEC)	Cattle and Buffalo	Genotypic (bla *Ctxm)*	Raw milk	27.3%	800	2022	(Ullah et al. 2022)

To counter the rising threat of AMR, the Ministry of National Health Services launched a National Action Plan (NAP) on AMR in May 2017 in Pakistan. According to this plan, various measures have been implemented at both the national and provincial levels (Saleem et al. 2022). However, there are still barriers to implementation, and the livestock sector continues to face weak enforcement, limited surveillance, and low awareness among farmers and veterinarians, contributing to high rates of AMU and AMR (Bilal et al. 2021; Farhan et al. 2024; Mohsin 2019; Saman et al. 2023). The 2016 Joint External Evaluation (JEE) conducted by the WHO highlighted an urgent need to expand AMR mitigation efforts, especially in veterinary and agricultural practices (World Health Organization 2017).

### c) Emergence of antimicrobial resistance in mastitis pathogens

The emergence of antibiotic-resistant pathogens in Pakistan, especially the methicillin-resistant and MDR *S. aureus* strains of bovine mastitis, is widely reported (Haq et al. 2024; Ijaz et al. 2023, 2024; Khan et al. 2022; Muzammil et al. 2022). Table 2 summarises the findings of the studies that reported the prevalence of resistant pathogens in Pakistan.

The data show a high and persistent prevalence of multidrug-resistant *S. aureus* (MDRSA) and methicillin-resistant *S. aureus* (MRSA) in dairy bovines across Pakistan (Table 2). MDRSA prevalence has reached 80% in buffaloes with CM (Khan et al. 2022), compared to 50% in cattle with SCM (Haq et al. 2024) and 20% in raw milk (Lubna et al. 2023), suggesting possible species-specific susceptibility, especially in clinical cases. MRSA was also detected at alarming levels, including 81.8% in raw milk (Lubna et al. 2023) and 50% to 74.3% in SCM cases (Aqib et al. 2018; Khan et al. 2019a, b, 2023; Ghumman et al. 2023). Studies from 2017 to 2024 consistently reported the widespread presence of the *mecA* gene, conferring methicillin resistance, with prevalence ranging between 17.0% and 74.2%, highlighting the extensive misuse of antibiotics and inadequate control measures. Additionally, vancomycin-resistant *S. aureus* (VRSA) showed a rising incidence, despite having relatively low prevalence (10.9%−12.6%) (Javed et al. 2021; Muzammil et al. 2023). Its emergence is alarming, given that vancomycin is a last-resort antibiotic and signifies a serious warning for potential treatment failures in critical cases (Wijesekara et al. 2017).

Resistance to other antibiotic classes is also widespread. Tetracycline resistance is notably high, with *tetK* detected in 63.64% of raw milk samples (Lubna et al. 2023) and over 50% resistance to *tetA*,* tetB*, and *tetC* genes in buffaloes with CM (Khan et al. 2022). Similarly, resistance to beta-lactam with *blaZ* was found in 55.32% of cattle SCM cases (Haq et al. 2024), and *blaTEM* in 63.9% of buffaloes with CM (Khan et al. 2022), highlights the declining efficacy of first-line antibiotics. Resistance to erythromycin (*ermA*: 53.1%) and sulphonamide (*Sul1*: 49.6%) was also reported in buffalo mastitis cases (Khan et al. 2022; Rasool et al. 2022). Though resistance to aminoglycoside (*aacA-aphD*: 13.83%) (Haq et al. 2024) was relatively low; its detection remains concerning. These trends reflect extensive, unregulated antibiotic use in dairy settings, limiting the therapeutic options and underscoring the urgent need for improved AMU practices and stewardship programs.

Multi-drug-resistant *K. pneumoniae* (44.4%) in cattle with CM (Saddam et al. 2023), and ESBL-producing *K. pneumoniae* (*blaSHV*; 56%) in SCM (Jamal et al. 2024) and Shiga toxin-producing *E. coli* (27.3%) in raw milk (Ullah et al. 2022) indicate the growing role of Gram-negative pathogens. This poses significant treatment challenges due to their intrinsic resistance mechanisms (Arzanlou et al. 2017) and the ability to transfer resistance genes horizontally (Durrani et al. 2024; Lerminiaux and Cameron 2019).

Notably, buffalo exhibits higher resistance to mastitis pathogens, especially in CM cases. A total of 80% MDRSA, 63.9% *blaTEM* (BRSA) and 52.5% *tetA* (TRSA) genotypically confirmed isolates were reported in buffaloes (Khan et al. 2022). This difference may be attributed to species-specific management and hygienic practices in buffaloes in Pakistan, such as rearing under smallholder, less intensive systems, where animals experience poorer sanitation, more frequent hand milking without proper teat disinfection, and longer treatment courses, all of which contribute to higher AMR in buffalo isolates. The increasing use of genotypic detection methods (Ghumman et al. 2023; Haq et al. 2024; Ijaz et al. 2024; Muzammil et al. 2023; Shahzad et al. 2024) reflects a shift towards more precise AMR surveillance, as reliance on phenotypic methods may create diagnostic uncertainties (Yee et al. 2021). The detection of resistant pathogens, including MDRSA, MRSA, TRSA, and ESBL STEC in raw milk (Lubna et al. 2023; Ullah et al. 2022) poses serious food safety risks due to their zoonotic potential, especially where unpasteurised milk is consumed.

## Current bovine mastitis management strategies in Pakistan

Despite decades of research, bovine mastitis remains a persistent challenge in Pakistan’s dairy sector (Shahzad et al. 2024). Management strategies primarily rely on antibiotic use and biosecurity measures, although implementation varies widely (Akhtar et al. 2023; Sharif et al. 2009). Generally, antibiotics are the most common intervention (Pascu et al. 2022), with aminoglycosides (197.52 mg/kg) being the most frequently used in Pakistan, followed by macrolides (171.1 mg/kg), tetracyclines (167.02 mg/kg), and sulphonamides (101.69 mg/kg) (Ullah 2023). Other antibiotics, such as polypeptides, quinolones, lincosamides, penicillins, and additional antibiotics are used in smaller quantities (Ullah 2023). For mastitis treatment specifically, aminoglycosides, beta-lactams, and tetracyclines are the preferred antibiotic classes (Mohsin and Umair 2020). However, the overuse of these antibiotics has led to increasing AMR, with studies reporting high resistance rates to gentamicin (aminoglycoside) and amoxicillin, and cefoxitin (beta-lactams) among mastitis pathogens (Ghumman et al. 2023; Muzammil et al. 2022). Beyond antibiotics, mastitis prevention strategies include improved milking hygiene, post-milking teat disinfection, selective dry cow therapy, and culling of chronically infected animals (Bilal et al. 2008; Kashif et al. 2016; Rehman et al. 2013; Sharif et al. 2009). However, limited farmer awareness, inconsistent adherence to biosecurity measures, and economic constraints hinder the widespread adoption of these practices (Nadeem and Ahmad 2024). Addressing these gaps through farmer education, improved diagnostic capabilities, and promoting non-antibiotic alternatives is essential to reducing reliance on antimicrobials and mitigating AMR risks in Pakistan’s dairy industry (Ashraf and Muhammad 2018; Ijaz et al. 2021; Jalil et al. 2022; Muzammil et al. 2022).

### a) Alternative treatment options for bovine mastitis in Pakistan

Various alternative treatment options have been tested against bovine mastitis pathogens in Pakistan (Table 3), including nutraceutical/non-antibiotic approaches (Ijaz et al. 2021), herbal/phytotherapy (Amber et al. 2018; Bilal et al. 2024; Khan et al. 2023; Naseer et al. 2021), nanoparticles (Aqib et al. 2021; Haider et al. 2020; Hannan et al. 2024; Nawaz et al. 2024; Ul-Hamid et al. 2023), probiotics (Hussain et al. 2021; Izhar et al. 2024, 2025), drug repurposing (Ahmad et al. 2023; Ahmed et al. 2022; Muzammil et al. 2022), bacteriophage therapy (Hamza et al. 2016; Najeeb et al. 2025), biological therapy (Ullah et al. 2024) and ethnoveterinary therapies (Dilshad et al. 2010). This reflects growing national interest in developing sustainable, residue-free and resistance-free treatments as alternatives to conventional antibiotics.

**Table 3:** Alternative treatment therapies in bovine mastitis in Pakistan

Category	Therapeutic agent	Study Type	Bovine (Species)	Target (SCM/CM)	Outcome	References
Nutraceutical/non-antibiotic	Trisodium citrate + Vitamin C + ZnSO₄ + CuSO₄	Experimental *(in vivo)*	Cattle	SCM	Significant reduction in SCC, improved milk pH, comparable efficacy to antibiotics, economic gain (Rs. 457/animal/day)	(Ijaz et al. 2021)
Herbal/Phytotherapy	Pistacia chinensis (fruit, bark, leaves); Cotoneaster microphyllus (leaves, bark)	In vitro	Cattle and Buffalo	SCM	Ethyl acetate bark extract of *P. chinensis* showed the highest inhibition (21.3 mm) against both MRSA and MSSA; moderate activity from *C. microphyllus* leaf extract	(Khan et al. 2023)
	Allium sativum, Bunium persicum, Oryza sativa, Triticum aestivum	In vitro	Cattle and Buffalo	Mastitis Pathogens (*E. coli*,* S. aureus*,* K. pneumoniae*, MDR strains)	Alkaloids and crude extracts of *(A) sativum* and *(B) persicum* showed strong antibacterial activity. Promising alternative therapies.	(Amber et al. 2018)
	Homeopathic Complex (Mastitojin Gold™) and Neem Seed Extract	In vivo (clinical trial)	Buffalo	SCM	Neem extract achieved an 80% cure rate; homeopathic complex 71%; both were less effective than antibiotics (91%) but cost-effective, with neem being the cheapest.	(Muhammad Younus et al. 2018)
	Azadirachta indica (Neem) methanolic leaf extract	In vitro (Disc diffusion)	Cattle	SCM	Neem extract showed better antibacterial efficacy than oxytetracycline, oxacillin, and cefoxitin.	(Bilal et al. 2024)
	Red chili (Capsicum annuum), Garlic (Allium sativum), Ginger (Zingiber officinale)	In vitro (agar well diffusion + MIC)	Buffalo	Mastitis Pathogens (MDR *S. aureus* and S. pyogenes*)	Red chilli had the strongest antibacterial effect individually (ZDI up to 24 mm, MIC as low as 0.211 mg/ml); red chilli + garlic showed the highest synergism, outperforming reference antibiotics.	(Naseer et al. 2021)
	Calotropis procera, Eucalyptus globulus + Amoxicillin	In vitro	Cattle and Buffalo	SCM (MRSA)	Plant extracts increased amoxicillin efficacy up to 31.29% (Calotropis). A combination of both extracts showed a significant synergistic antibacterial effect.	(Aqib et al. 2021)
Nanotechnology	Tungsten oxide (WO₃) nanoparticles + Ciprofloxacin	In vitro (Disc diffusion + MIC)	Cattle and Buffalo	SCM	WO₃-ciprofloxacin combination significantly enhanced antibacterial activity vs. resistant *E. coli* and *S. aureus*. *E. coli* showed greater sensitivity. MIC values reduced notably over time.	(Nawaz et al. 2024)
	Phytochemically reduced NiO nanoparticles using Zingiber officinale (ginger) and Allium sativum (garlic)	In vitro (agar diffusion)	Cattle	Mastitis Pathogen (MDR *S aureus)*	Garlic-mediated NiO NPs showed stronger inhibition (5.9 mm at 1.0 mg/50 µl) than ginger and outperformed crude extracts; ciprofloxacin was used as a reference control (12.55 mm).	(Haider et al. 2020)
	ZnO and Zn(OH)₂ + Amoxicillin	In vitro	Cattle and Buffalo	SCM (MRSA)	ZnO microparticles increased amoxicillin ZOI by 26.74%; Zn(OH)₂ by 14.85%. Modulation factors indicated strong synergy.	(Aqib et al. 2021)
	Biologically synthesized TiO₂ (ginger & garlic)	In vitro	Cattle	MDR *S. aureus* (Mastitis Origin)	Garlic-reduced TiO₂ showed a strong bactericidal effect against MDR strains; docking confirmed binding to bacterial enzymes	(Ul-Hamid et al. 2023)
	Ciprofloxacin-loaded Cerium Oxide/Chitosan nanocarrier (CIP-CeO₂/CS)	In vitro	Cattle	MRSA (Mastitis Origin)	The CIP-CeO₂/CS nanocarrier showed enhanced antibacterial activity at a lower MIC (8 mg/mL) compared to free CIP, was hemocompatible, and provided sustained drug release.	(Zafar et al. 2021)
Probiotic Therapy	*Lactobacillus plantarum* strain CM49	In vitro (Cell line) In vivo (Mice Model)	Cattle	Mastitis pathogens *(S. aureus*, *E. coli*, *Strep. dysgalactiae)*	*Lactobacillus plantarum* showed strong antibacterial activity in vitro, significant auto/co-aggregation, and reduced mastitogen growth in broth. It inhibited pathogen adhesion on bovine mammary epithelial cells and reduced mastitis severity in mouse models. Safe with no acquired antibiotic resistance.	(Izhar et al. 2024)
	*Lactobacillus plantarum* strain CM49	In vivo (Cattle)	Cattle	SCM and CM	Intramammary infusion of CM49 for 5 days increased lactobacilli counts, enhanced microbial diversity, and reduced *Streptococcus* in clinical mastitis and *Staphylococcus* in subclinical mastitis.	(Izhar et al. 2025)
	Cell-free supernatants (organic acids) from *Lactobacillus delbrueckii*,* Enterococcus faecium*,* Weissella confusa*	In vitro	Cattle and Buffalo	MDR *S. aureus*, *E. coli*, *Salmonella Typhi*, *P. aeruginosa*	CFS showed strong inhibition via organic acids (lactic, acetic, propionic); strains were non-hemolytic, antibiotic-sensitive, acid/bile-tolerant, suitable for probiotic use	(Hussain et al. 2021)
Drug Repurposing	Gentamicin + Ketoprofen Sulfamethoxazole + Meloxicam Oxytetracycline + Meloxicam	In-vitro + In-vivo (rabbit model for MRSA skin infection)	Cattle and Buffalo	MRSA (Mastitis Origin)	Significant synergistic effects observed with NSAID + antibiotic combinations. Gentamicin + ketoprofen showed the highest cure rate. Zones of inhibition improved significantly with combinations, supporting resistance modulation potential.	(Muzammil et al. 2022)
	NSAIDs (Flunixin, Meloxicam, Ketoprofen), Ivermectin + Amoxicillin, Gentamicin, Cotrimoxazole	In vitro	Cattle	MDR & biofilm-positive *S. aureus*	Combinations of NSAIDs with antibiotics (e.g., flunixin + gentamicin) showed synergistic effects (FICI ≤ 0.5).	(Ahmed et al. 2022)
	Tylosin (IM), Gentamycin (IM/M), Combination (Tylosin + Gentamycin)	In vivo (clinical trial)	Buffalo	CM (*S. aureus*)	Combination therapy had the highest cure rates: 80.76% clinical and 76.92% bacteriological at 28 days post-treatment. IM alone had better clinical cure than the local route alone.	(Ahmad et al. 2023)
Bacteriophage Therapy	SA Bacteriophage (lytic phage)	In vitro	Cattle and Buffalo	*S. aureus* (Mastitis origin)	Phage SA showed high lytic activity against MDR *S. aureus* strains	(Hamza et al. 2016)
	Phage UHP46	Experimental/Genomic	Cattle	*MDR S. aureus* (mastitis origin)	Phage UHP46 showed strong lytic activity against MDR *S. aureus*, good stability at 20–45 °C and pH 6–8, and no resistance or virulence genes	(Najeeb et al. 2025)
Biological	Autologous Platelet-Rich Plasma (PRP)	In vivo (Clinical Trial)	Buffalo	SCM	PRP alone reduced SCC more effectively than antibiotics or a combination. PRP was 9.67x more effective than the antibiotic alone, and 55% more productive than PRP + antibiotic.	(Ullah et al. 2024)
Ethnoveterinary	25 local plant species (e.g., Capsicum annuum, Allium sativum, Curcuma longa, Zingiber officinale, etc.) + ammonium chloride	Field survey	Cattle and Buffalo	CM and SCM	Locals used combinations of indigenous herbs and substances orally or topically. The most frequent remedies involved *Capsicum annuum*, *Lepidium sativum*, *Allium sativum*, and *Citrus limon*.	(Dilshad et al. 2010)

*S. pyogenes is not a typical mastitis pathogen in buffaloes in Pakistan. Its mention here follows Naseer et al., (2021), where it was reported in mixed infections alongside S. aureus, and is included solely to demonstrate alternative therapy efficacy

Herbal and probiotic therapies are the most widely studied, with various in vivo and in vitro trials (Amber et al. 2018; Bilal et al. 2024; Izhar et al. 2024). Phytotherapies show promising results due to their low incidence of adverse effects, low cost, lesser drug residues in animals and the environment and are less prone to the development of antibiotic resistance (Lopes et al. 2020). Plants such as *Pistacia chinensis*,* Allium sativum*, *Bunium persicum*, *Azadirachta indica*, and *Capsicum annuum* have demonstrated strong antibacterial effects against multidrug-resistant pathogens of mastitis origin (Amber et al. 2018; Bilal et al. 2024; Khan et al. 2023; Naseer et al. 2021). Probiotic strains, including *Lactobacillus plantarum CM49* and postbiotics from other lactic acid-producing bacteria (LAB), have shown strong pathogen inhibition, immune modulation, and safe use profiles against drug-resistant pathogens of bovine mastitis origin (Hussain et al. 2021; Izhar et al. 2025) as shown in Table 3.

Nanoparticle-based therapies and NSAID-antibiotic synergy testing (drug repurposing) have shown enhanced antibacterial potential by modulating resistance against mastitis pathogens. Studies have reported increased zones of inhibition and reduced MIC values, especially when NSAIDs combined with antibiotics (Ahmed et al. 2022; Muzammil et al. 2022). Nanotechnology-related approaches have also shown increased antibacterial efficacy against MDR mastitis pathogens, particularly when used in combination with conventional antibiotics (Aqib et al. 2021; Haider et al. 2020; Nawaz et al. 2024).

Bacteriophage therapy, particularly against *S. aureus*, has also been reported to significantly reduce bacterial growth and holds potential as an alternative therapy (Hamza et al. 2016; Najeeb et al. 2025). In rural Pakistan, many farmers rely on ethnoveterinary practices for treating bovine mastitis. Among 217 traditional veterinary healers surveyed, the most commonly used plants included *Capsicum annuum*, *Lepidium sativum*, *Allium sativum*, and *Sesamum indicum* as alternative treatment options (Dilshad et al. 2010). Despite the encouraging outcomes, most studies remain at the in vitro stage. Although several alternative treatments show promising in vitro and in vivo results, most lack standardised protocols, comprehensive safety assessments, and large-scale field validation in Pakistan, which limits their immediate applicability. There is a critical need for standardisation of treatment protocols, safety evaluation and in-field validation of these alternative treatments within the context of Pakistan’s dairy sector.

### b) Management and biosecurity practices for mastitis prevention

Different management and biosecurity practices, such as hygiene, milking practices, and housing conditions, also play an important role in the occurrence of bovine mastitis (Yaqoob 2019). A study by Maalik et al. (2019) found that bovine SCM incidence was lower (26.3%) in animals where pre-milking teat disinfection was practised, compared to 53.8% in animals without teat disinfection, and mastitis was more common in cows on hard brick floors compared to those on soft sand bedding. Studies have shown a higher incidence of bovine mastitis on farms with hand milking (Khan et al. 2019a, b), poor hygiene practices (Ali et al. 2021), delayed diagnosis, poor ventilation, inadequate housing conditions (Hussain et al. 2012), and a lack of dry cow therapy implementation (Kashif et al. 2016).

### c) Government interventions

Following the endorsement of the Global Action Plan to tackle AMR at the 68th World Health Assembly in Geneva in May 2015, Pakistan, under the supervision of the Ministry of National Health Services, Regulations & Coordination (NHSR&C), developed and implemented the ‘National Strategic Framework for Containment of Antimicrobial Resistance’ in 2016. Incorporating a one health approach across human health, veterinary and agriculture sectors, the framework was integrated into the National Action Plan (NAP) to curb AMR (NHSR&C et al. 2017). The main strategic priorities of the NAP include the development and implementation of a comprehensive national strategy to address AMR through awareness, surveillance, infection control, regulatory enforcement, research integration, alternatives to growth promoters, and burden estimation. Under the AMR steering committee in 2018, Pakistan developed Multisectoral Coordination Committees, in which provincial focal points for AMR have been appointed within the livestock department (Saleem et al. 2022). To strengthen surveillance, two national reference laboratories have been designated as AMR reference labs (National Institutes of Health 2022). However, the overall surveillance framework remains fragmented, which reduces its impact. Pakistan has also integrated AMR into veterinary curricula, conducted awareness training, reported AMU data to WOAH since 2016 and banned critical antibiotics as growth promoters in Punjab. However, implementation remains limited due to weak enforcement, lack of funding, and insufficient awareness among stakeholders (Qiu et al. 2024). These policies demonstrate commitment at the national level. However, their effectiveness is constrained by fragmented implementation, limited enforcement, insufficient funding, and low awareness among farmers and local veterinarians.

## Future perspectives and recommendations

To improve mastitis management and reduce the risk of AMR in Pakistan’s dairy sector, the following policy-level recommendations are proposed. These recommendations are intended for government and regulatory bodies, rather than practical, farm-level solutions for producers. These recommendations offer practical guidance. However, their implementation may face challenges due to variability in local infrastructure, farmer engagement, and resource availability. They focus on creating a supportive framework that empowers farmers with simple, affordable tools and knowledge, encourages community collaboration, and promotes sustainable practices that can be integrated into small-scale farming operations in Pakistan.

1. Facilitate frequent low-cost consultations between local veterinarians and the farmers involving simple pathogen detection and prevention, and reduce reliance on costly antibiotics.
2. Establish antimicrobial stewardship programs that veterinary officers or farmer groups run to educate farmers about the dangers of antibiotic misuse and advise how to treat the diseases without overdosing.
3. Improve the early detection of mastitis with simple diagnostic tools such as visual observation or locally fabricated test kits to lessen the dependency on expensive machines. Train the farmers in conducting these tests.
4. Enforce regulatory measures of antibiotic consumption, including the ban of over-the-counter sales and low-cost veterinary provision for proper treatment.
5. Promote simple hygiene, biosecurity, and low-cost alternative cures, including herb remedies, which small-scale farmers can buy at subsidised costs or through cooperatives.
6. Train farmers on mastitis prevention through electronic media, mobile phones and community programs with clear but locally understandable relevant content.
7. Create programs educating farmers on treating with antibiotics and diagnosing mastitis using cost-effective tools.
8. Provide ongoing training for local veterinarians and community health workers, focusing on practical skills for passing on to farmers.
9. Improve local AMR surveillance and provide farmers with easy ways to report issues like mastitis-related problems, including cases, unusual signs and treatment failure using mobile phones or veterinary officers.
10. Direct funding towards research on low-cost alternatives to antibiotics, including natural remedies and farm-specific or cooperative vaccine initiatives, to help manage mastitis and AMR.

## Limitations

This review presents a broad overview of bovine mastitis and AMR in Pakistan, but some limitations must be acknowledged. Firstly, most of the available studies are regional or based on limited sample sizes, which may constrain their applicability across the diverse dairy systems of Pakistan. Secondly, there is no strong nationwide data on antibiotic use patterns and resistance rate trends, particularly among the smallholders. Thirdly, most of the available literature is observational or descriptive, and there are few longitudinal or intervention-based studies that can be used to support causality or long-term outcomes. Moreover, although other practices like herbal remedies are mentioned, they must be more scientifically proven to be effective and safe. This review focuses on bovine mastitis and AMR in Pakistan. Comparisons with neighbouring countries or global averages were not included, but could be explored in future studies to provide a broader context. The above limitations dictate that more directed studies should be conducted, and national surveillance and field-based assessments need to be done to guide evidence-based policies.

## Conclusion

This review explored the prevalence of bovine mastitis, antimicrobial usage patterns, emerging resistance pathogens, economic impact, and management strategies to control mastitis within Pakistan’s dairy sector. Despite having a considerable dairy population and production potential, most of the issues encountered in the dairy industry revolve around AMR, inappropriate use of antibiotics, and poor farm management. This overuse of essential antibiotics in most cases without veterinary supervision resulted in the emergence of resistant pathogens, which threaten not only livestock’s health but also public health. Moreover, the lack of a comprehensive economic model has led to minimal understanding by all dairy farmers regarding the actual cost of mastitis. To curb this issue, this review proposes a regular consultation with veterinarians at a low cost, promoting regionally specific diagnostic equipment and educational programs, including antimicrobial stewardship. Regulatory interventions are required, like controlling the sale of over-the-counter antibiotics, encouraging biosecurity and hygiene at farms to prevent diseases, and facilitating research projects on devising alternative treatment options for bovine mastitis. Such interventions would help reduce the reliance on antibiotics and bring about sustainable and smallholder-friendly control of mastitis in Pakistan. However, knowledge gaps exist, especially in assessing the on-farm economic losses and the practical feasibility of proposed interventions under varied local conditions. Additional studies need to be conducted on AMR surveillance, disease control strategies, farmers’ awareness, and therapeutic strategies to create a more sustainable dairy industry in Pakistan.

## Data Availability

The datasets generated during and/or analysed during the current study are available from the corresponding author on reasonable request.
